# Management of the premaxilla in the treatment of bilateral cleft of lip and palate: what can the literature tell us?

**DOI:** 10.1007/s00784-015-1589-y

**Published:** 2015-09-16

**Authors:** Gerhard K. P. Bittermann, Ad P. de Ruiter, Nard G. Janssen, Arnold J. N. Bittermann, Aebele M. van der Molen, Robert J. J. van Es, Antoine J. W. P. Rosenberg, R. Koole

**Affiliations:** Department of Oral and Maxillofacial Surgery, University Medical Centre Utrecht, G05.129, Heidelberglaan 100, Utrecht, 3584 CX Utrecht The Netherlands; Department of Otorhinolaryngology–Head and Neck Surgery, University Medical Center Utrecht, Utrecht, The Netherlands; Department of Plastic Surgery, University Medical Center Utrecht, Utrecht, The Netherlands

**Keywords:** BCLP, Premaxilla, Osteotomy, Alveolar bone grafting

## Abstract

**Objective:**

In the treatment of bilateral cleft lip and palate (BCLP) patients, there is discussion about the management of the position of the premaxilla. This literature analysis summarises the literature on managing this condition.

**Materials and methods:**

A PubMed, Embase and Cochrane Library search was conducted resulting in 4465 articles which were screened on title and abstract.

**Results:**

Seventy-one articles were available in full text, 16 of which were included in this literature analysis. We searched on keywords timing and technique, complications, growth of the maxilla and results after bone grafting the alveolar process. This literature analysis has shown that there are various ways to correct the position of the premaxilla. These can be divided into primary, early, late secondary and tertiary intervention before the age of 8 years, between the ages of 8 and 12 years and older than 12 years. Correction is done with surgery, orthodontics or a combination, with or without bone grafting.

**Conclusions:**

An osteotomy of the premaxilla in combination with secondary alveolar bone grafting appears to be the most successful technique. Combining early secondary alveolar bone grafting with osteotomy creates more room to ensure a watertight closure of the nasal mucosa resulting in fewer postoperative complications. Before surgery, the orthodontist should try to optimise the position of the premaxilla for its surgical correction prior to bone grafting.

**Clinical relevance:**

The treatment of BCLP patients is still based on experience and expert opinions. This literature analysis tries to give a summery on how to handle the protruded and displaced premaxilla.

## Introduction

Patients with bilateral cleft lip and palate (BCLP) require much attention. Between prenatal diagnosis at 20 weeks of pregnancy and the birth of a child with a cleft, it cannot be taken for granted that the parents will accept the child [1]. Up to the age of 18, children and young people with BLCP require intensive treatment in the fields of diet, growth, psychosocial development, hearing and cosmetic disorders [2].

An important aspect of the BCLP patient is that the alveolar clefts cause the premaxilla to be mobile from birth and only apically fixed to the vomer bone. The premaxilla is often protruding due to the lack of sphincter function of the orbicularis oris muscle. This causes extreme abnormalities in the position of the premaxilla; sometimes, the whole segment is rotated and functional and cosmetic disorders result. Over time, there have been many forms of treatment aimed at changing the position of the premaxilla. In the past, the premaxilla was sometimes resected [3]. Later, early osteotomy of the premaxilla was carried out (setback osteotomy) during or even before lip closure. This had a disastrous effect on the growth of the maxilla [4, 5]. In a review of the treatment results of BCLP patients, Vargervik et al. highlighted severe growth disturbances in 12 patients treated by early surgery [6]. Since then, the prevailing opinion has been that carrying out osteotomy of the premaxilla before the age of 6 years should be avoided [7].

In patients with BCLP, closure of the alveolar clefts is usually carried out at a later stage—between the ages of 9–12 years—and involves a bone graft and a corrective osteotomy of the maxilla [8–11]. The aims of this literature review were to collect data on the position of the premaxilla and the correction of the malposition in the areas of (a) timing and technique, (b) stability of the position achieved and the remaining alveolar bone volume, (c) the effects of surgery on maxillary growth and (d) complications reported in the literature.

## Materials and methods

### Search protocol and selection of articles

#### Search and selection

**Fig. 1:**
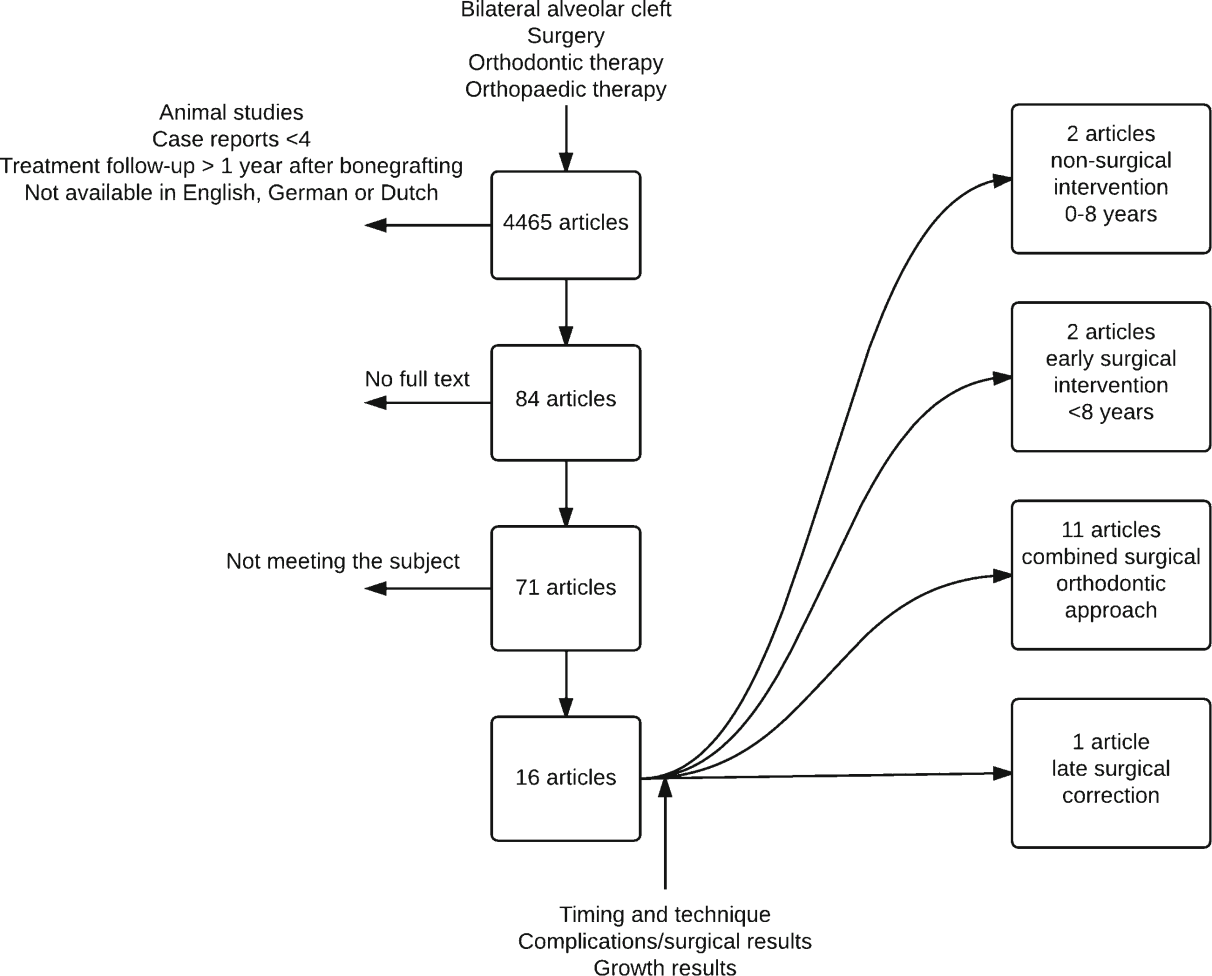
Schematic record of the search process: review articles and references from selected articles were manually screened for titles not identified during the initial search

A systematic search in PubMed, Embase and the Cochrane Library was conducted covering the period from 1960 to January 2015. The search terms ‘bilateral cleft lip and palate’, ‘premaxilla osteotomy’, ‘surgery’, ‘orthodontics’, orthopedics, ‘secondary alveolar bone grafting’ and ‘bilateral alveolar cleft’ and all relevant synonyms were used (Table 1) [12]. Only those articles written in English and German were collected in this literature search. Using predefined inclusion and exclusion criteria, one author (GB) screened all retrieved articles on title and abstract and excluded duplicate titles to select potentially eligible articles. Inclusion criteria were availability of full text and case studies containing groups of four patients or more with details of follow-up to premaxilla osteotomy and describing the success and the complications of premaxilla osteotomy. Subsequently, the full text of relevant articles was screened for further selection. Finally, review articles on this topic and references from selected articles were manually screened for titles not identified during the initial search (Fig. 1). All articles describing nasoalveolar moulding (NAM) were excluded, as this procedure is an early technique that takes place before lip closure.

**Table 1:** Keywords used for the search of the three databases

Database	Terms
PubMed	((((((((((((((“bilateral alveolar cleft” [Title/Abstract]) OR “bilateral alveolar clefts” [Title/Abstract]) OR “secondary alveolar bone grafting” [Title/Abstract]) OR “blcp” [Title/Abstract]) OR “alveolar cleft” [Title/Abstract]) OR “alveolar clefts” [Title/Abstract]) OR “premaxilla” [Title/Abstract]) OR “premaxillary” [Title/Abstract]) OR “bilateral cleft” [Title/Abstract]) OR “bilateral cleft alveolus” [Title/Abstract]) OR “bilateral cleft lip/cleft palate” [Title/Abstract]) OR “intermaxillare” [Title/Abstract]) OR ((“Cleft Palate/surgery” [Mesh] OR “Cleft Palate/therapy” [Mesh]))))
	AND
	((((((((((((((((((surgery[Title/Abstract]) OR “surgical” [Title/Abstract]) OR “surgeries” [Title/Abstract]) OR “operation” [Title/Abstract]) OR “operated” [Title/Abstract]) OR “operate” [Title/Abstract]) OR “reposition” [Title/Abstract]) OR “repositioning” [Title/Abstract]) OR “repositioned” [Title/Abstract]) OR “graft” [Title/Abstract]) OR “grafted”) OR “grafting” [Title/Abstract]) OR “surgically” [Title/Abstract]) OR “orthodontic” [Title/Abstract]) OR “orthodontically” [Title/Abstract]) OR “orthodontics” [Title/Abstract]) OR “orthopedics” [Title/Abstract]) OR “orthopedic” [Title/Abstract])
EMBASE	(‘bilateral alveolar cleft’:ab,ti OR ‘bilateral alveolar clefts’:ab,ti OR ‘secondary alveolar bone grafting’:ab,ti OR bclp:ab,ti OR ‘alveolar cleft’:ab,ti OR ‘alveolar clefts’:ab,ti OR premaxilla:ab,ti OR premaxillary:ab,ti OR ‘bilateral cleft’:ab,ti OR ‘bilateral cleft alveolus’:ab,ti OR ‘bilateral lip cleft palate’:ab,ti OR intermaxillare:ab,ti OR ‘cleft palate’/exp)
	AND
	(surgery:ab,ti OR surgical:ab,ti OR surgeries:ab,ti OR operation:ab,ti OR operated:ab,ti OR operate:ab,ti OR reposition:ab,ti OR repositioning:ab,ti OR repositioned:ab,ti OR graft:ab,ti OR grafted:ab,ti OR grafting:ab,ti OR surgically:ab,ti OR surgically:ab,ti OR orthodontic:ab,ti OR orthodontically:ab,ti OR orthodontics:ab,ti OR orthopedics:ab,ti OR orthopedic:ab,ti) AND [embase]/lim NOT[medline]/lim
Cochrane	Cleft palate mesh

## Results

The results of the literature search are summarised in Tables 2 and 3. After screening, 16 articles were included in this analysis. These included two articles concerning non-surgical interventions, 2 articles concerning early surgical interventions, 11 articles on combined orthodontic and surgical therapy and 1 concerning a late surgical intervention.

**Table 2:** Summary of the articles included in this literature analysis

Author (reference)	Patients cases	Intervention	Follow-up	Premaxilla position	Other
Scott [1]	15 Retrospective follow-ups	Secondary alveolar bone grafting + osteotomy	3 Years	–	93.96 % of bone height is retained after 3 months
Geraedts [2]	40 Retrospective follow-ups	Early secondary alveolar bone grafting + osteotomy	8 Years	At age 17, 13 patients needed osteotomy	Cephalometric analysis
					Le Fort needed in nine patients
Brouns [3]	31 Retrospective follow-ups	Osteotomy + bone graft, sometimes second operation	1 Year	Angle class I/II group, 17 patients good occlusion	Bigger position corrections result in bigger chance of complications
				Angle class III group, 13 patients good occlusion	
				Angle class IV group, one patient good occlusion	
Carlini [4]	50 Retrospective follow-ups	Secondary alveolar bone grafting + osteotomy	1 Year	–	–
Freihofer [5]	13 Retrospective follow-ups	Secondary alveolar bone grafting + osteotomy	15 months	–	Residual bone height in 15 patients more than 50 %
Heidbuchel [6]	22 Retrospective follow-ups	Secondary alveolar bone grafting + osteotomy	7 Years	SNA average increase 2.02°	18 Patients more than 50 % maxilla height.
				3.34° reduction of angle between spinal plane and SN plane	Cephalometric analysis
Grabowski [7]	18 Follow-ups	Orthopaedics and orthodontics (osteoplasty)	17.3 Years	Nine patients, SOB >2 mm, VOB >2 mm	Cephalometric results
					Criteria: time of treatment, type of orthodontic treatment, method of closing the incisor gap in the cleft area, special methods.
				Seven patients, SOB >3 mm	
				VOB >3 mm	
Cronin [8]	5 Case studies	Osteotomy and Kirschner wire fixation	–	–	–
Liou [9]	8 Prospective follow-ups	Tooth-borne distraction device	1 Year	Before treatment, SOB average 8.4 mm, VOB 3.7 mm	46 % true orthopaedic intrusion of the premaxilla, 54 % dental intrusion of the premaxilla
				After treatment, SOB 0.7 mm	
Padwa [10]	24 Non-randomised controlled trials	Early vs. late vs. no osteotomy of the premaxilla	2.8–5.7 Years	–	Cephalometric results
Akita [11]	17 Non-randomised controlled trials	Osteotomy of the premaxilla with secondary alveolar bone grafting vs. secondary alveolar bone grafting alone	0.5–3 years	–	0–25 % bone resorption in the osteotomised group in 12/14 clefts.
					0–25 % bone resorption in the non-osteotomised group in 10/20 clefts
Bishara [12]	20 Retrospective follow-up controlled studies	Premaxilla osteotomy at time of lip closure vs. two-staged closure of the lip and no osteotomy	17 years	–	Cephalometric analysis
Koh [13]	8 Retrospective case studies	Interdental distraction osteogenesis, alveolar bone grafting, premaxilla osteotomy	56 months	–	Bone height, 98 % of patients between 50 and 100 % left
Aburezq [14]	4 Retrospective follow-ups	Secondary alveolar bone grafting + osteotomy	10 months	–	Two patient → grade 1
					One patient → grade 2
					One patient → grade 3
Oyama [15]	6 Case studies	Secondary alveolar bone grafting + osteotomy	3 months	–	Bone height is good
Narayanan [16]	14 Case studies	Palatal repair and premaxilla setback	6 months	10–15 mm setback. Five had class III relationship. Two had open bite	Good results

*SOB* sagittal overbite, *VOB* vertical overbite

**Table 3:** An overview of the scored parameters collected from the selected literature

Article	Outcome parameter	Comment
Timing and technique of premaxillary correction		
Grabowski et al. (2006)	Orthopaedic and orthodontic treatment starting at an early age:	
	*N* = 18 Good results no osteotomy required.	
	Two patients with crossbite	
	Class III in three patients	
Liou et al. (2004)	Orthopaedic and orthodontic treatment between 8 and 11 years:	
	*N* = 8 correction of the premaxilla using a distractor	
	Overbite reduction to 0.7 mm	
Cronin et al. (1957)	Surgery during neonatal period	Bad outcome for maxillary growth
Bishara et al. (1972)	Osteotomy during neonatal period, compared with no osteotomy:	Early osteotomy has a bad outcome on maxillary growth
	SNA significantly smaller in osteotomy group.	
	SNB negative	
	Concave soft tissue profile	
Heidbüchel et al. (1993)	Orthodontics and osteotomy:	After osteotomy, better inclination of maxillary incisors
	*N* = 22 prior to surgery orthodontic intrusion of the premaxilla. After osteotomy, SNA decreased by 2.02	
Scott et al. (2007)	Age 8–12 years, good results surgical correction of the premaxilla.	Collagen membrane was used to close the nasal mucosal layer
Koh et al. (2013)	*N* = 51, 36 patients treated with bone grafting only.	
	*N* = 7 wide cleft, good premaxillary position. Treated with a distractor	
	*N* = 8 surgical repositioning of premaxilla.	
Brouns et al. (1980)	*N* = 31 surgical repositioning of premaxilla	
	In the Angle class I and II groups, good occlusion	
	In the Angle class III group, good occlusion	
	In the class IV group, good occlusion	
Akita et al. (2006)	*N* = 17 divided into two groups	
	*N* = 10 no premaxillary osteotomy	
	*N* = 7 premaxillary osteotomy	
Aburezq et al. (2006)	*N* = 4 osteotomy combined with secondary alveolar bone grafting.	
Freihofer et al. (1991)	*N* = 13 surgical repositioning of the premaxilla between 8 and 12 years	
	*N* = 8 preoperative orthodontics	
	*N* = 10 postoperative orthodontics	
Geraedts et al. (2007)	*N* = 40 combination of pre-orthodontic treatment and repositioning of the premaxilla between 8 and 12 years	
Narayanan et al. (2006)	Tertiary osteotomy in children in developing countries.	Children were not operated on until the tertiary osteotomy
Stability of the position of the premaxilla and bone volume		
Scott et al. (2007)	*N* = 15 iliac crest bone transplants, all successful	
	93.96 % bone volume preserved after 3 months	
Koh et al. (2013)	In 96.1 % of patients, more than 50 % transplant bone volume was preserved	
Brouns et al. (Brouns & Egyedi, (1980)	Of the Angle class I and II patients *N* = 17, *N* = 11, consolidation no premaxillary instability	
	Angle class III group *N* = 13, *N* = 11 good consolidation	
	*N* = 2 remaining unstable premaxilla	
	Class IV *N* = 1 group stable premaxilla	
Carlini et al. (2009)	*N* = 50, 45 patients no mobility of the premaxilla.	
Akita et al. (2006)	The amount of bone required to fill the cleft was significantly lower in the osteotomy group.	
Aburezq et al. (2006)	*N* = 3 with good consolidation and more than 50 % bone volume left.	After trauma
	*N* = 1 unstable premaxilla	
Freihofer et al. (1991)	*N* = 9 rib bone	
	*N* = 3 mandibular bone	
	*N* = 1 local bone	
	*N* = 12 premaxilla stable and more than 50 % of bone preserved	
Narayanan et al. (2006)	Uninhibited growth up to time of surgery on the premaxilla.	
Effects of surgery or orthodontic intervention on maxillary growth		
Cronin et al. (1957)	Surgery during neonatal period with disastrous effect on growth	
Bishara et al. (1972)	Surgery during neonatal period with bad effect on maxillary growth	
Geraedts et al. (2007)	*N* = 27 acceptable profile at the end of follow-up	
	*N* = 13 hypoplastic midface for which a Le Fort I procedure was carried out	
	No significant differences between osteotomy and non-osteotomy groups	
Padwa et al. (1991)	*N* = 24 comparing three groups, for the effect of age on midfacial growth at time of surgery. Youngest group 6 years old.	
	No delay in midfacial growth in any of the groups.	
Complications reported in the literature		
Heidbüchel et al. (1993)	Premaxillary necrosis in one patient (5 %)	
Scott et al. (2007)	*N* = 15, three patients with wound dehiscence	
Brouns et al. (1980)	In the class III group, 11 patients with residual fistula, no necrosis of the premaxilla	
Carlini et al. (2009)	*N* = 50 successful premaxilla repositioning and bone grafting in 48 patients. Two patients with premaxillary necrosis.	
Aburezq et al. (2006)	No necrosis of the premaxilla. One patient with residual fistula	
Freihofer et al. (1991)	*N* = 1 necrosis of the bone transplants on both sides	
Geraedts et al. (2007)	*N* = 1 recurrent oronasal fistula	

The literature shows that there are three periods of time during which the position of the premaxilla can be corrected.

1. Early primary correction
  - Early non-surgical correction during the first year of life and <8 years
  - Early surgical correction <8 years
2. Early and late secondary combined treatment (between 8 and 12 years)
3. Late surgical correction (>12 years tertiary)

For each of these treatment periods, we focused on analysing the following:

- The position and stability of the premaxilla and the results of bone grafting to the clefts.
- The effects of this treatment on the growth of the maxilla.
- The complications reported in the selected literature

### Early non-surgical correction during the first year of life and <8 years

During the first year of life, the position of the premaxilla can be guided by nasoalveolar moulding (NAM) or pre-surgical orthodontic treatment (PSOT) [13]. The application of NAM or PSOT prior to surgery is to enable easier primary lip closure between the age of 0 and 12 months. However, the long-term results of these techniques on the position of the premaxilla are not reported in the literature [14]. As this is an early therapy used before closure of the lip and the long-term results on the position of the premaxilla are not clear, NAM and PSOT were excluded from this review [15].

After closure of the lip and reconstruction of the muscle orbicularis oris, a short-term growth effect is seen in the maxilla [16].

Dentofacial orthopaedic and orthodontic procedures are also carried out. Of the articles that were finally selected, two described the use of dentofacial orthopaedic and orthodontic procedures [17, 18].

Grabowski et al. [17] describe the long-term follow-up (17.3 years) of 18 patients who underwent orthopaedic and orthodontic interventions without osteotomy. Pont’s Index was used to measure the width of the dental arch in BCLP patients with permanent dentition [19]. Lateral cephalogram was used to determine the overjet. In 4 of the 18 patients, high anterior compression of the upper jaw developed following treatment. Two patients developed a crossbite, and an Angle class III jaw relationship was found in three patients. At the age of 17, two patients had a sagittal overbite and a vertical overbite of 0 mm. Nine patients had a sagittal overbite of more than 2 and 3 mm, and seven had a vertical overbite of more than 2 and 3 mm. It was not necessary to widen the upper jaw or carry out orthognathic surgery in any of these patients. Guiding the growth of the bone during the first year of life results in more space in the upper jaw and fewer extractions later in life. Before 1990, clefts were not closed; after 1990, an osteoplasty was carried out.

Liou et al. [18] describe eight patients between the ages of 8 and 11 during the mixed dentition period with caudally prominent premaxilla, in whom the average overbite was 8.4 mm and the average vertical overbite was 3.7 mm. These patients were treated with a dentally fixed distractor on the premaxilla and affixed to the upper molars which moved the intruding premaxilla cranially. In the whole group, intrusion was complete within 4 weeks. The overbite was significantly reduced to 0.7 mm. After the intrusion, the occlusion surface and the gingival margins of the premaxilla and the lateral segments were level.

### Early primary surgical correction <8 years

In the 19–50s, surgical correction or resection of the premaxilla was carried out at an early stage. This took place during the closure of the lip or in the period thereafter. Two articles from the literature search report on the use of this technique [20, 4].

Cronin et al. [20] described cases of a large protrusion where an osteotomy of the premaxilla was carried out during the neonatal period to improve lip closure. Pre-vomerine resection was carried out. The premaxilla was moved dorsally, and the vomer bone obtained in this way was put into the clefts. In the 19—70s, a comparable study was produced by Bishara et al. [4]. They looked at the cephalometric differences between patients in whom an osteotomy of the premaxilla had been carried out at the time of lip closure and at patients in whom the premaxilla had not been manipulated. In the osteotomy group, the age at which this had been carried out was 2.5 months; in the non-osteotomy group, the lip had been closed in two stages. The first operation was at the age of 2.5 months and the second between 2.5 and 6 months. The average age at evaluation was 18.6 years in the osteotomy group and 17.2 years in the non-osteotomy group. At this age, midfacial growth is complete. In the osteotomy group, the sella-nasion-A point (SNA) angle was found to be significantly smaller. This shows an unfavourable effect on the ventral growth of the maxilla. Unlike the non-osteotomy group, the A point-nasion-B point (ANB) angle was negative which led to the conclusion that there was an unfavourable effect on growth. The soft tissue profile of the osteotomy group was concave in shape, whereas that of the non-osteotomy group was convex in shape. On measurement of the position of the mandible, there proved to be no significant differences between the groups.

### Early and late secondary combined treatment (between 8 and 12 years)

Combined treatment involves optimising the position of the premaxilla by means of orthodontic treatment followed by osteotomy of the premaxilla and bone grafting. Eleven articles from the literature search describe this method [21–24, 9, 25–27, 7, 28, 29, 2].

The article of Heidbüchel et al. [29] describes the combination of orthodontic and surgical treatment of BCLP patients with a follow-up of 7 years. There were 22 patients included in the study. Prior to surgery, the premaxilla was positioned cranial of the occlusion surface in five patients and at the same level or below the occlusional surface in 17 patients. In two cases, the premaxilla was already in class III relationship. Preoperatively, the upper jaw was widened in 12 patients, and the upper incisors had to be aligned in five patients. In one patient (5 %), the premaxilla was lost after the osteotomy due to necrosis. On cephalometry, the SNA angle was defined as a measure of the protrusion of the premaxilla. The SNA decreased by an average of 2.02° following osteotomy, and there was a reduction of 3.34° in the angle between the spinal plane and the sella-nasion (SN) plane. The angle between the upper incisors and the SN plane increased by 14.34°, resulting in a more normal inclination of the maxillary incisors.

Scott et al. describe 15 patients with a follow-up of 3 years who underwent osteotomy of the premaxilla and bone grafting from the iliac crest [28]. All the bone grafts were successful, and no necrosis of the premaxilla was observed. In three patients, there was dehiscence of the wound which was treated conservatively. At 3 months, the average bone height was 93.96 %, and at 3 years, 79 % of canines had erupted in the bone graft. Collagen membrane was used to close the nasal mucosal layer, and in combination with an osteotomy of the premaxilla, this ensured good closure nasally.

Koh et al. [21] found the position of the premaxilla to be acceptable in 36 of 51 patients treated only with an alveolar cleft closure by bone grafting from the iliac crest. In seven patients who had a very wide cleft and in whom the premaxilla was well positioned, the two halves of the jaw were drawn towards each other by means of a distractor to make the cleft smaller (Erverdi et al. 2012). In the other eight patients, the position of the premaxilla was unfavourable and an osteotomy of the premaxilla was carried out. An alveolar bone graft was done at a separate procedure. The position of the premaxilla was regarded as unfavourable if the horizontal overbite (SOB) was more than 9 mm or less than −3.5 mm. In 96.1 % of patients, more than 50 % of the bone graft was preserved (Abyhölm grades 1–2) [30].

Brouns et al. [24] describe a corrective osteotomy in 31 BCLP patients. They repositioned the premaxilla, and in some cases, they also carried out an osteotomy of the lateral alveolar process. If there was adequate bone contact, no bone was grafted from the iliac crest. In some cases, this was done at a later time. The patients were divided into class 1 to class IV premaxilla-mandible relationship. Class IV is an Angle class III relationship with the premaxilla in a cranial position (front open bite). In the Angle class I + II groups (*N* = 17), the premaxilla was well consolidated in 11 patients. In the other six patients, there was persisting premaxillary instability. All 17 patients had good lateral occlusion. In the Angle class III group (*n* = 13), consolidation achieved by a bone graft was good in 11 patients, and in 13 patients, occlusion was good. The remaining two patients had persistent instability of the premaxilla. In the class III group, 11 patients had a residual fistula. In the class IV group (*n* = 1), consolidation and occlusion were both good. There was no necrosis of the premaxilla.

Carlini et al. [9] describe 50 patients in whom surgical repositioning of the premaxilla was carried out in combination with a bone graft. In 24 patients, bone from the mandibular symphysis was used for grafting, and in 26 patients, bone was harvested from the iliac crest. The operation was successful in 48 (96 %) patients, but 2 patients developed necrosis of the premaxilla. In 45 of the 48 patients, there was no mobility of the premaxilla postoperatively; therefore, good consolidation had been achieved. There was some bone loss in three of the remaining patients (6 %), but after a second operation, treatment was successful. No difference was found between the bone from the mandibular symphysis and the bone from the iliac crest in the alveolar cleft closure procedure.

Akita et al. [23] describe a comparison between two groups of patients. The first group had a less-pronounced abnormality of the premaxilla (*n* = 10), and no osteotomy of the premaxilla was carried out; in the second group who had a more pronounced abnormality (*n* = 7), this procedure was carried out. An osteotomy of the premaxilla was combined with a bone graft from the iliac crest. The amount of bone required to fill the cleft properly was significantly lower in the osteotomised group. There was also significantly less bone resorption in the osteotomy group.

In their article, Aburezq et al. [22] describe four patients who were treated with an osteotomy of the premaxilla combined with secondary alveolar bone grafting. There was no loss of the premaxilla, and good consolidation was seen in three patients. In these patients, there was less than 50 % resorption of the grafted bone [30]. Following a trauma, postoperative instability of the premaxilla developed in one patient. This patient also developed a unilateral fistula and an infection. On the side of the fistula, bone height was below 50 %, and on the contralateral side, it was above 50 %. All patients had a well-aligned dental arch.

Freihofer et al. [25] describe 13 BCLP patients aged between 8 and 12 years who were treated with an osteotomy of the premaxilla. Eight of these patients also underwent preoperative orthodontic treatment, and ten underwent postoperative orthodontic treatment. Rib bone was used in nine patients and chin bone in three patients. In one case, there was enough local bone to close the cleft. Of the 24 oronasal fistulas, 22 were closed and there were two recurrences—both in the same patient. In 24 clefts, a bone bridge developed; in one patient, this remained absent bilaterally. In 12 patients, the premaxilla remained stable. In one patient (8 %), the bone grafts were resorbed bilaterally and the premaxilla became necrotic. In the other 12 patients, grafted bone made up more than 50 % of the height of the maxilla.

As, when carrying out an osteotomy of the premaxilla, it is possible to damage the growth centre, it is important to know what the long-term results are and pay special attention to growth. Geraedts et al. [26] describe the long-term follow-up of early secondary closure in combination with an osteotomy of the premaxilla in 40 patients between 8 and 12 years old. Rib bone was used in 11 patients and chin bone in 25 patients. In four patients, only vomer bone was used. In 17 patients, a pharyngoplasty was carried out at the age of 5–6 years, and in 4 patients, a Le Fort I osteotomy was done at the age of 18 years. One patient developed a recurrent oronasal fistula. The facial profile was acceptable in 27 of the 40 patients, and the sagittal and vertical dental relationships were essentially correct. Of these 40 patients, 13 had a hypoplastic midfacial deformity for which they underwent a Le Fort 1 osteotomy. Nine other patients were offered a Le Fort I osteotomy, but they did not want to undergo further surgery. In the group with a non-acceptable profile, no further operations such as pharyngoplasty and secondary nose correction were carried out. This study used a control group of patients who did not undergo osteotomy of the premaxilla, and there were no significant differences between the groups.

Padwa et al. [7] did extensive research into midfacial growth following osteotomy of the premaxilla. This study compared 24 patients divided into three groups: 7 underwent osteotomy of the premaxilla before the age of 8 years (6.1 years), 10 were over the age 8 years (11.2 years), and 7 did not undergo osteotomy. When the preoperatively measured SNA and SNB angles of each of the groups were compared, it was shown that there was more anteposition and nasal rotation of the premaxilla in the early group. For this reason, the movement of the premaxilla during the osteotomy was largest in this group. However, at the final postoperative check-up, there were no significant differences in the position of the premaxilla between the groups; i.e. no delay of growth was measured in any of the groups.

### Late surgical correction (tertiary >12 years)

Late surgical correction is mainly carried out in developing countries where patients often only present with bilateral clefts at a later age. Uninhibited growth is possible until an older age. The literature search produced one article in which this is described [31].

In summarising the results of the literature, we focused on the following items

#### The position of the premaxilla and the results of bone grafting

The selected articles describe a total of 259 osteotomies of the premaxilla. The complications and results of 121 of these procedures are clearly described. In 100 patients, the premaxilla was stable, and in 121 patients, more than 50 % of the grafted bone was still present. Of this group of 259 patients, 81 patients underwent autologous bone grafting from the iliac crest, 38 from the mandibular symphysis, 20 from the ribs and 3 from local bone. In the remainder, the donor site is not reported. Total necrosis and loss of the premaxilla are described in four of these patients. Some of the selected articles reported the results of the premaxilla osteotomy and secondary alveolar bone grafting. Very few data are available on recurrent fistulas (Table 4). The aim of carrying out an osteotomy of the premaxilla is to improve its position.

**Table 4:** Summary of complication rates collected from the articles selected for this review

Article	Number of patients	Complications described in article	Type bone graft (*N*)	Complication (*N*)	Patients with stable premaxilla	Less than 50 % bone graft resorption
Scott et al. (2007)	15	15	Iliac crest (15)	Wound dehiscence (3)	12	15
Brouns et al. (1980)	31	31	Iliac crest (31)	No consolidation (9)	22	22
Carlini et al. (2009)	50	50	Iliac crest (26)/mandibular symphysis (24)	Bone loss (3)/premaxillary necrosis (2)	45	45
Freihofer et al. (1991)	13	13	Rib bone (9)/mandibular symphysis (3)/other (1)	Premaxillary necrosis (1)	12	12
Cronin et al. (1957)	40	–	–	–	–	–
Heidbuchel et al. (1993)	22	1	Rib (11)/iliac crest (5)/mandibular symphysis (4)/maxillary (1)/bank bone (1)	Premaxillary necrosis (1)	–	18
Padwa et al. (1999)	17	–	–	–	–	–
Akita et al. (2006)	7	7	Mandibular symphysis (7)	Bone loss (1)	6	6
Bishara et al. (1972)	20	–	–	–	–	–
Aburezq et al. (2006)	4	4	Iliac crest(4)	Bone loss (1)	3	3
Geraedts et al. (2007)	40	–	–	–	–	–
Total	259	121	142	20	100	121

*N* number of patients

#### The effects of treatment on the growth of the maxilla

The selected articles [20, 4] describe the effect of early surgical intervention on growth of the premaxilla. It can be concluded from these articles that it is very disadvantageous for midfacial growth to undergo surgery to correct the position of the premaxilla before the age of 6 years. Selected articles describe the effects of an osteotomy of the premaxilla on midfacial growth at a later age. These articles report that there do not appear to be any significant differences in the results if an osteotomy is or is not carried out [7, 26].

#### Summary of complications reported in the literature

Of the 11 selected articles that describe surgical intervention to correct the position of the premaxilla, 7 report the occurrence of complications. These range from dehiscence of the wound, recurrent fistulas, loss of grafted bone due to resorption and instability of the premaxilla to complete necrosis and loss of the premaxilla. Table 4 summarises the complications described in the selected articles.

## Discussion

In BCLP patients, the position of the premaxilla can be very abnormal [32]. This malposition could be a sagittal Angle class III jaw relationship or a class I or II division crossbite jaw relationship, in both cases with a large variation in the vertical relationship with the mandibular frontal teeth. The premaxilla may also be in torsion. This wide variety in presentation occurs because the connection with the septo-premaxillary ligament is the factor that determines the direction of growth. The direction of growth is also determined by pressure from the tongue and lip [24, 33].

### Early primary correction before 8 years non-surgical

This type of correction using orthopaedic and orthodontic procedures achieves good results. Even earlier NAM instigated directly after birth makes primary lip closure between 0 and 12 months easier [15]. However, the results of this have also been called into question in the literature [14, 34]. At a slightly older age, it is possible to carry out orthodontic procedures that influence the position of the premaxilla and the width of the upper jaw [35].

Orthopaedic interventions are used to guide the growth of the jaw from birth. A number of articles describe how to use growth to influence the position of the upper jaw and the premaxilla. This results in a great improvement in the position of the premaxilla; often, osteotomy is no longer necessary. It is important to create good occlusion as soon as possible after the permanent dentition has erupted. While the patient still has deciduous teeth, orthodontics can be used to regularise the position of the premaxilla. In this way, growth can be better guided [17]. The application of orthodontics at a young age requires an expert approach which focuses on oral hygiene and guidance [36].

### Early surgical correction before 8 years and the effects of timing of surgery on growth

One of the areas from where the upper jaw grows is the premaxillary vomerine suture which is the site of osteotomy of the premaxilla [26, 7, 37]. This can potentially result in damage to this growth centre and retardation of growth at a later age. Growth from this centre is responsible for the forward and vertical growth of the entire midface [4, 16]. From the literature, it appears that if an osteotomy of the premaxilla is carried out at a very early age (2.5 months at the same time as lip closure), retardation of midfacial growth can occur [4]. This should be taken into account, and an osteotomy of the premaxilla should be carried out after the age of 6 years when 90 % of midfacial growth is complete [4, 7].

The long-term follow-up of patients treated solely by orthodontics or orthopaedics shows that few growth problems are to be expected [38, 16, 39].

### Early and late secondary combined treatment (between 8 and 12 years)

By far, the majority of articles describe combined treatment whereby the position of the premaxilla is corrected by orthodontic intervention before osteotomy of the premaxilla is carried out. As well as the premaxilla being in a good anatomical position, the continuity of the alveolar process is also relevant. Eleven of the selected articles describe this premise. However, the timing of the operation and the way in which it is carried out differ between studies. In order to achieve an uninterrupted dental arch, bone is grafted to both sides of the premaxilla. The canines or the lateral incisors will be able to erupt into the newly formed bone or can be moved there by orthodontic treatment. The methods and timing of this vary. Current opinion is that early or late secondary alveolar bone grafting should be carried out between the ages of 9 and 11 years, prior to the eruption of the permanent upper canines and when the root has reached one third to one half of its final length. If the permanent lateral incisors are present at a younger age, then of course, this should be carried out earlier, between the ages of 7 and 9 years [40, 41, 29, 26, 37, 4].

A bone graft can be carried out in combination with an osteotomy of the premaxilla or at a separate session following the osteotomy. Without an osteotomy of the premaxilla, the clefts can also be closed in one or two stages [21]. The underlying philosophy is that if large bilateral defects need to be filled, it is better to do so in two stages (Kamakura et al. 2003). However, this is rarely done as, normally, there is more than enough bone to fill both sides of the defect. There are some clinical circumstances that may force the surgeon to interrupt the surgical procedure, for example, ischaemia of the premaxilla. The common goal is to perform the osteotomy and bone grafting at the same procedure.

### Complications and results of bone grafting

The complication that is mentioned in practically all the articles is loss of grafted bone both unilaterally and bilaterally due to infection or dehiscence of the wound (Table 4). Recurrent instability of the premaxilla and recurrent oronasal fistulas is also mentioned. The most severe complication is necrosis and loss of the premaxilla due to compromised circulation in the buccal pedicle [9].

A long-term complication of osteotomy of the premaxilla is retardation of the growth of the upper jaw due to damage to the vomerine growth centre of the upper jaw.

## Conclusion

With or without osteotomy of the premaxilla—with or without bone graft—all the authors in this literature search have their own preferences and techniques for the treatment of BCLP patients. There appears to be no common opinion. The treatment of patients with a bilateral cleft differs both internationally and between centres. Current treatment protocols are based on retrospective studies and expert opinion. The consensus of opinion is that alveolar bone grafting and osteotomy of the premaxilla should preferably be done at one session at around the age of 8 years or older. In the opinion of this review, carrying out an osteotomy of the premaxilla after the age of 8 years has more advantages. However, it is also our opinion that only after all orthodontic methods have been exhausted should there be an indication for carrying out osteotomy of the premaxilla. Bone grafting of the clefts is carried out at the same time as the osteotomy [25]. Surgical treatment in combination with secondary alveolar bone grafting has many advantages. The canines will erupt in the correct position ensuring that minimal prosthetic rehabilitation is required. Surgical correction in a vertical direction is more difficult than it is in a posterior, anterior or transverse direction [7].

If a vertical overbite of more than +4 mm or a vertical open bite of more than −2 mm is measured, an osteotomy of the premaxilla is justified. This applies to every negative sagittal relationship to the premaxilla and to the reverse torque position and if the premaxilla is rotated (axis 11 in relation to an SN of less than 100°).

The literature shows in the matters of (a) premaxilla position and bone height, (b) timing of surgery and growth and (c) reported complications that an osteotomy of the premaxilla should always be considered in combination with (and at the time of) early secondary alveolar bone grafting (8–12 years). This will give the best result in these three categories.

At the Wilhelmina Children’s Hospital cleft centre (Utrecht, NL), the carrying out of the osteotomy and bone grafting in one procedure has generally been found to be technically difficult, but good clinical results are achievable.
